# Effects of registered nurse staffing levels, work environment, and education levels on adverse events in nursing homes

**DOI:** 10.1038/s41598-021-00946-8

**Published:** 2021-11-02

**Authors:** Seonhwa Choi, Eunhee Cho, Eunkyo Kim, Kyongeun Lee, Soo Jung Chang

**Affiliations:** 1 College of Nursing, Yonsei University, 50-1, Yonsei-ro, Seodaemoon-Gu, Seoul, 03722 South Korea; 2 Mo-Im Kim Nursing Research Institute, Yonsei University College of Nursing, 50-1, Yonsei-ro, Seodaemoon-Gu, Seoul, 03722 South Korea; 3 Department of Nursing, Tongmyong University, 428, Sinseon-ro, Nam-gu, Busan, 48520, South Korea; 4 Department of Nursing, Gangneung-Wonju National University, 150, Namwon-ro, Heungeop-myeon, Wonju, 26403 South Korea

**Keywords:** Health care, Health occupations

## Abstract

This study examined the effects of nurse staffing levels, work environment, and education levels on adverse events in nursing homes. A cross-sectional study was conducted involving 216 nurses working in 62 nursing homes in South Korea, using self-reported questionnaires and data from the National Health Insurance Service of South Korea. A logistic regression model was used to investigate the effects of nurse staffing levels, work environment, and nursing education levels on the adverse events experienced by residents. An increase of one resident per nurse was significantly associated with a higher incidence of medication error, pressure ulcers and urinary tract infections. A poor work environment increased the incidence of adverse events. Compared to nurses with a bachelor’s degree or higher, those with diplomas reported increased incidence rates of pressure ulcers. Improving the health outcomes of residents in nursing homes requires efforts that strengthen the nursing workforce in terms of numbers and educational level, and which improve their work environment at institutional and policy levels.

## Introduction

The pace of population aging around the world is increasing dramatically due to decreasing birth and mortality rates^1^. This rapid population aging means that strategies should be implemented at national and global levels immediately. The World Health Organization has developed five global strategies on aging and health. One of these strategies, “strengthen long-term care,” involves establishing a long-term care system and continuously improving it, reinforcing the long-term care workforce, helping informal caregivers, and enhancing and maintaining the quality of integrated and person-centered long-term care^1^. Similarly, ensuring quality of care and an appropriate nursing workforce for residents in nursing homes are global concern.

In South Korea, the Long-Term Care Insurance system, which has been operating since 2008, covers senior citizens older than 65 years or senior citizens not yet 65 years old with geriatric diseases^2^. Under the influence of this insurance system, the number of nursing homes has increased from 1700 in 2008 to 5360 in 2019^2^. Most nursing home operating agents are private owners (72.6%), followed by corporations (25.1%) and local governments (2.1%)^2^. Although people have more choices for a nursing home through various operating agencies, there are differences in quality of care as well as regional distribution gaps among nursing homes^3^. Most residents of nursing homes not only have cognitive or functional restrictions caused by chronic progressive diseases but also have more comorbid diseases compared to community-dwelling seniors who function well^4,5^. In addition, they may be at high-risk of being exposed to infectious disease outbreaks due to their grouped living conditions^6^. Therefore, nursing home residents have specific and demanding healthcare, management and treatment needs that require appropriate nursing^4,5^.

A registered nurse (RN) is the only healthcare professional working in nursing homes in South Korea^7^. They play an essential role in nursing homes, such as managing other nursing personnel and caregivers, coordinating care work, conducting close observation of residents, communicating and cooperating with medical staff, monitoring infection control, conducting wound care, and running programs to improve quality management^8^. However, certified nursing assistants (CNAs) can replace RNs according to South Korea’s employee placement policy^9^. There were 3147 RNs in 5360 Korean nursing homes and 11,332 CNAs as of June 2019^2^. This means that, from the perspective of those eligible for admission, the quality of healthcare services that they receive may vary depending on which nursing home they enter^5^.

According to a report on the adequacy of nurse staffing in hospitals and nursing homes by the Institute of Medicine’s (IOM) Committee, nursing is a crucial factor in determining the quality of care and health-related outcomes of patients^10^. In seeking to provide empirical evidence concerning this matter, many researchers have examined the effects of nurse staffing on care quality or nursing home residents’ health-related outcomes, including safety-focused outcomes, in the United States and Europe^10–12^.

However, the correlations found between nurse staff and the quality of care or residents’ health outcomes were not consistent across previous studies and reviews^11–15^. Spilsbury and colleagues, based on their study results, pointed out that previous research had focused on measuring quality in relation to the numbers of RN and suggested measuring the association between other staffing characteristic factors and quality of care in future research^12^. The quality of care provided by nurses is influenced by their professional characteristics such as education and experience as well as nursing systems, which are related to staffing levels, and by the workplace environment such as the physical environment, communication systems and collaboration, and relevant support services^10,12^.

Residents’ health outcomes in nursing home care refer to the end results, including changes in health status and conditions that can be attributed to the amount of care provided^12^. Adverse events such as medication errors, falls with injury, urinary tract infection, pneumonia, and pressure ulcers are considered as important health outcomes^16^; however, these adverse events have not been sufficiently studied in Korean nursing homes. Recently, in South Korea, research has been conducted on nurse staffing and quality of care in nursing homes^7,17,18^. However, few studies have examined the effects of RN staffing levels, including educational characteristics and perceived work environment, on adverse events in nursing homes in South Korea. Therefore, this study aimed to examine the effects of RN staffing levels, their work environment, and their education levels on adverse events experienced by residents in nursing homes.

## Methods

### Design, setting, and participants

In this cross-sectional study, contact was made with all nursing homes with 100 or more beds (*n* = 107) where at least one RN worked, in Seoul, Gyeonggi province, and 6 metropolitan cities in South Korea. Out of 107 nursing homes, 62 agreed to participate in this study. The institutional characteristics of the participating nursing homes were determined using data concerning long-term care facilities published on the National Health Insurance Service website of South Korea. The subsequent process and the purpose, methods, and procedures of this study were explained to nurses working in the participating nursing homes and written informed consent was obtained from all participants. The survey was administered to 224 nurses who agreed to participate in the study. The inclusion criteria were employment at a nursing home and currently working for more than 3 months. A research team distributed questionnaires to the nurses, and the nurses answered the questionnaires in a confidential and separate area such as a conference room in the nursing homes; sealed questionnaires were collected by the research team. If a nurse was not working on the day of data collection or was working on night shift, a written research explanation, research consent form, questionnaire, and return envelope were left to be collected by these nurses. In such cases, the study purpose was explained by phone and, to maintain confidentiality, the nurses involved were asked to answer the questionnaire in a separate area and send back the sealed questionnaires directly to the research team. Data were collected from April 2016 to July 2016. Questionnaires from 219 nurses in 62 nursing homes were returned, and 3 uncompleted questionnaires were excluded from the data analysis. Finally, questionnaires from 216 nurses in 62 nursing homes were included for data analysis. This study received ethical approval from the Institutional Review Board of Yonsei University College of Nursing (No. YUCON-IRB-2015-0024) and was performed in accordance with IRB regulations according to the Declaration of Helsinki.

### Measures

#### Dependent variables

Adverse events, which were considered as dependent variables, included medication errors, pressure ulcers, sepsis, falls with injury, urinary tract infections and pneumonia. In South Korea, patient electronic charts are not used in most nursing homes and there is no reporting system for adverse events and patient health outcomes in nursing homes; thus, survey data collected by nurses were used to identify adverse events in this study. Nurses were asked to rate the frequency (in terms of “never,” “a few times a year or less,” “once a month or less,” “a few times a month,” “once a week,” “a few times a week,” and “every day”) of adverse events that occurred among older residents they were caring for.

#### Independent variables

The characteristics of the nursing homes included location (Seoul as the capital city of Korea vs. Gyeonggi province and 6 metropolitan cities), the number of beds (100–199 vs. 200 or above), ownership (local government vs. corporation vs. private), and the number of RNs per facility (continuous variable). The general characteristics of the RNs included gender, age, working years as an RN and education (diploma vs. bachelor’s degree or higher). Nurse staffing levels were surveyed in terms of the number of residents who were assigned to the RNs on the last shift at the time of the survey. The nurse-reported work environment was measured using the Practice Environment Scale of the Nursing Working Index (PES-NWI) in nursing homes^19^. The PES-NWI in nursing homes consists of 27 items across five domains. The five domains include: (1) nurses’ participation in nursing home affairs (6 items); (2) well-defined scope of practice (4 items); (3) nurse managers’ ability, leadership, and support of nurses (7 items); (4) staff and resource adequacy (3 items); and (5) communication and coordination (7 items). In the study of Cho et al., Cronbach’s ⍺ for the total 27-item instrument was .93, and for the domains, it ranged from .77 to .88^19^. In this study, Cronbach’s ⍺ for the total items was .93. The score was calculated according to the method presented in the study of Cho et al.^19^. For a generalized measure of the practice environment, the mean of each domain in each nursing homes was compared with the median of each domain from all the nursing homes and, for each nursing home, the number of domains was counted that were above the median for all the nursing homes. If the number of domains for each nursing home with a higher mean than the median of the whole domain was 4–5, the nurses’ work environment was categorized as “better;” if it was 2–3, it was categorized as “mixed;” and if it was 0–1, it was categorized as “poor”^19^.

### Data analysis

The collected data were analyzed using SPSS for Windows (version 25.0). Descriptive statistics and logistic regression were used for data analysis. The general characteristics of the nursing homes, the nurses, and the adverse events the nurses reported were analyzed using descriptive statistics. A logistic regression model was used to control for the characteristics of the nursing homes (location, ownership, and number of beds) and the RNs (age and work experience) as well as to examine the effects of nurse staffing levels (number of older adults assigned to an RN), work environment (the PES-NWI in nursing homes) and the level of nursing education on the adverse events experienced by residents. The numbers of medication errors and pressure ulcers, and sepsis frequency, were analyzed using binaries, with “never” indicating zero and “a few times a year or less” to “every day” indicating one. However, for falls with injury, urinary tract infections and pneumonia, because the frequencies of “never” were too small, the analysis was carried out using binaries, with “never” and “a few times a year or less” indicating zero and “once a month or less” to “every day” indicating one.

## Results

### Characteristics of the nursing homes

The general characteristics of the 62 nursing homes are shown in Table 1. Of the total 62 nursing homes, 18 (29.0%) were located in Seoul. Moreover, 51 nursing homes had 100 to 199 beds (82.3%), and 11 nursing homes had 200 or more beds (17.7%). In terms of establishment type, 10 nursing homes (16.1%) were operated by local governments, 44 (71.0%) were operated by corporations, and 8 (12.9%) were operated by individuals. The average number of RNs per nursing home was 3.84 ± 3.72 with a range of 1–16 RNs. Twenty-three nursing homes (37.1%) had poor work environments, 19 nursing homes (30.7%) had mixed work environments, and 20 nursing homes (32.3%) had better work environments.

**Table 1:** Characteristics of nursing homes (N = 62) and RNs (N = 216).

Characteristics	Category	N (%) or M ± SD (range)
**Nursing homes (*n* = 62)**		
Location	Seoul (capital)	18 (29.0)
	Others	44 (71.0)
Number of beds	200 or above	11 (17.7)
	100–199	51 (82.3)
Ownership	Local government	10 (16.1)
	Corporation	44 (71.0)
	Private	8 (12.9)
Number of RNs per facility		3.84 ± 3.72 (1–16)
Work environment	Poor	23 (37.1)
	Mixed	19 (30.7)
	Better	20 (32.3)
**RNs (*n* = 216)**		
Gender	Female	215 (99.5)
	Male	1 (0.5)
Age (years)		48.13 ± 9.43 (26–66)
Work experience as an RN	Years	15.36 ± 8.28 (0.58–40.75)
Education	Diploma	125 (57.9)
	Bachelor or higher	91 (42.1)
Number of older residents per nurse per shift		62.28 ± 42.60 (6–274)

*RNs* Registered nurses.

### Characteristics of the RNs

A total of 219 RNs answered the questionnaire. In the analysis, responses from three RNs were excluded because of missing data, and responses from 216 RNs were included. Most of the RNs were female (n = 215, 99.5%) with a mean age of 48.13 ± 9.43 years and a mean of 15.36 ± 8.28 years of work experience as a nurse. Ninety-one (42.1%) RNs had a bachelor’s degree or higher. The number of older residents cared for by an RN showed a mean of 62.28 ± 42.60, with a range from 6 to 274 older residents per shift (Table 1).

### Frequencies of adverse events

Table 2 provides information on the frequencies of adverse events reported by the RNs in the nursing homes. In the context of types of adverse events, 121 (56.0%) RNs reported medication errors as a few times a year or less, and 8 (3.7%) RNs reported such events as once a month or less. There were 136 (63.0%) RNs who reported pressure ulcers as a few times a year or less, and 8 (3.7%) RNs reported such events once as a month or less. Eighty (37.0%) RNs reported sepsis as a few times a year or less. Most of the RNs (n = 173, 80.1%) reported falls with injury as a few times a year or less, and 19 (8.8%) RNs reported such events as a few times a month. In the case of urinary tract infections, 168 RNs (77.8%) reported such events as a few times a year or less, and 18 RNs (8.3%) reported such events as once a month or less. Finally, in the case of pneumonia, 168 (77.8%) RNs reported such events as a few times a year or less, and 22 (10.2%) RNs reported such events as once a month or less.

**Table 2:** Frequencies of adverse events self-reported by RNs in nursing homes (N = 216).

Event frequency *n* (%)^†^	Types of adverse events					
	Medication errors (missing n = 7)	Pressure ulcers (missing n = 3)	Sepsis (missing n = 10)	Falls with injury (missing n = 5)	Urinary tract infections (missing n = 8)	Pneumonia (missing n = 2)
Never	73 (33.8)	67 (31.0)	125 (57.9)	8 (3.7)	7 (3.2)	5 (2.3)
A few times a year or less	121 (56.0)	136 (63.0)	80 (37.0)	173 (80.1)	168 (77.8)	168 (77.8)
Once a month or less	8 (3.7)	8 (3.7)	1 (0.5)	10 (4.6)	18 (8.3)	22 (10.2)
A few times a month	4 (1.9)	2 (0.9)	0 (0.0)	19 (8.8)	13 (6.0)	18 (8.3)
Once a week	2 (0.9)	0 (0.0)	0 (0.0)	0 (0.0)	0 (0.0)	1 (0.5)
A few times a week	0 (0.0)	0 (0.0)	0 (0.0)	1 (0.5)	2 (0.9)	0 (0.0)
Every day	1 (0.5)	0 (0.0)	0 (0.0)	0 (0.0)	0 (0.0)	0 (0.0)

^†^Numbers may not sum up to total sample due to missing data. Percentages also may not sum up to 100% due to missing data.

*RNs* Registered nurses.

### Effects of RN staffing levels, work environment, and education levels on adverse events in the nursing homes

Table 3 provides the results of the logistic regression model for the associations of RN staffing levels, work environment, and education levels with adverse events (medication errors, pressure ulcers, sepsis, falls with injury, urinary tract infections and pneumonia) in nursing homes while controlling for nursing home characteristics (location, ownership, and number of beds) and RN characteristics (age and work experience). Gender was not included in the logistic regressions since there was only one male participant. The models separately examined the effects of RN staffing levels, their work environment (the PES-NWI in nursing homes) and their education levels on each adverse event. An increase of one resident per RN was significantly associated with a higher incidence of medication errors [odds ratio (OR) = 1.009, 95% confidence interval (CI) = 1.002–1.017], a higher incidence of pressure ulcers (OR = 1.009, 95% CI = 1.001–1.017) and a higher incidence of urinary tract infections (OR = 1.009, 95% CI = 1.001–1.017). Compared to “better work environment”, “poor work environment” increased the incidences of pressure ulcers (OR = 9.748, 95% CI = 3.165–30.018), the incidences of sepsis (OR = 6.824, 95% CI = 2.365–19.685), falls with injury (OR = 12.729, 95% CI = 2.287–70.584), the incidences of urinary tract infections (OR = 3.803, 95% CI = 1.153–12.547) and the incidences of pneumonia (OR = 4.042, 95% CI = 1.301–12.555). Compared to RNs with a bachelor’s degree or higher, those with diploma degrees reported increased incidence rates of pressure ulcers (OR = 3.746, 95% CI = 1.680–8.353).

**Table 3:** Logistic regression model for adverse events (N = 216).

Variable	Medication errors	Pressure ulcers	Sepsis	Falls with injury	Urinary tract infections	Pneumonia
	OR (95% CI)	OR (95% CI)	OR (95% CI)	OR (95% CI)	OR (95% CI)	OR (95% CI)
Nurse staffing level	1.009 (1.002–1.017)*	1.009 (1.001–1.017)*	1.005 (0.998–1.011)	1.003 (0.994–1.012)	1.009 (1.001–1.017)*	1.002 (0.995–1.010)
**PES-NWI (Ref: better)**						
Mixed	1.109 (0.397–3.102)	0.945 (0.319–2.801)	2.832 (0.874–9.180)	1.617 (0.119–22.051)	1.133 (0.219–5.874)	1.714 (0.407–7.218)
Poor	1.675 (0.662–4.239)	9.748 (3.165–30.018)***	6.824 (2.365–19.685)***	12.729 (2.287–70.854)**	3.803 (1.153–12.547)*	4.042 (1.301–12.555)*
**Education level (Ref: BSN or higher)**						
Diploma	0.617 (0.310–1.230)	3.746 (1.680–8.353)**	1.594 (0.791–3.210)	0.417 (0.154–1.129)	1.260 (0.482–3.293)	0.876 (0.359–2.140)

This analysis controls for nursing home characteristics (location, ownership & number of beds) and nurses’ characteristics (age and work experience). The PES-NWI (Practice Environment Scale of the Nursing Working Index) for a mixed work environment showed no statistically significant relationship with all independent variables. The number of medication errors and pressure ulcers, and sepsis frequency, were analyzed as binaries, with “Never” indicating (0) and “A few times a year or less ~ everyday” indicating (1). However, because the “Never” frequency of falls with injury, urinary tract infections and pneumonia was too small, the analysis was conducted as binaries, with “Never and a few times a year or less” indicating (0) and “Once a month or less ~ everyday” indicating (1).

*OR* Odds ratio, *95% CI* 95% confidence interval, *Ref* Reference, *BSN* Bachelor of Science in Nursing.

*p < 0.05; **p < 0.01; ***p < 0.001.

## Discussion

The purpose of this study was to examine the effects of RN staffing levels, their work environment, and their education levels on adverse events experienced by residents in nursing homes. First, this study found that a higher RN staffing level was significantly associated with a lower incidence of medication errors, pressure ulcers and urinary tract infections among residents. These results indicate that RN staffing levels are an important factor influencing the likelihood of these specific adverse events and are consistent with previous research results suggesting that RN staffing levels affect preventable adverse events^13,17,20,21^.

However, other adverse events such as sepsis, falls with injury, and pneumonia were not influenced by the number of RNs. As a possible explanation, this finding may be related to the characteristics of RN staffing in South Korean nursing homes. According to White et al., in attempting to understand why the correlation between RN staffing and quality has been inconsistent, some nursing homes are so understaffed that small increases in the numbers of RNs do not lead to more substantial supervision of residents^8^. In South Korea, the legally required nursing staffing standard for nursing homes is 1 RN or CNA per 25 residents. However, the ratio of RNs among all employees in nursing homes has been reported to be only 1:1000, which indicates a lower RN standard than that of the United States^22^. Moreover, although the qualifications and roles differ between RNs and CNAs, CNAs can replace RNs as prescribed by the law^7^. For this reason, the work environment or salaries of RNs are little different from those of CNAs in many South Korean nursing homes, and the turnover rate of RNs is high^7^. Consequently, as seen in Table 1, in most nursing homes, the actual number of residents under one RN’s care per shift can be as high as 62.28 ± 42.60. This also means that the allotted time for residents’ direct care provided by the RN is likely to be limited. This situation would appear to explain why RN staffing levels did not show significant effects on three adverse events in this study. Nevertheless, the study showed a significant effect of RN staffing levels on lowering incidences of medication errors, pressure ulcers and urinary tract infections, and provides further supporting evidence for the importance of appropriate RN staffing levels in relation to reducing or preventing adverse events.

Second, the effect of the RNs’ work environment was also investigated concerning six adverse events in nursing homes. This study found that the prevalence of 5 adverse events, excluding medication errors, was 3.80 to 12.73 times higher when reported by RNs who perceived their work environment as poor rather than as better. These findings showed that a poor work environment for RNs was significantly associated with adverse events, which are similar to the results of previous studies^8,23–26^. According to Lim et al.^27^, the ratio of one RN to older adults who qualify for long-term care service has increased 2.3 times, from 1:79.35 in 2008 to 1:223.68 in 2018, which means that the workload of RNs has increased 2.3 times between 2008 and 2018 due to the decreased number of RNs working in South Korean nursing homes. Moreover, they reported problems such as role ambiguity between RNs and CNAs, absence of practical training programs tailored to the situation of nursing homes, low salaries, and communication or decision-making conflicts with directors of nursing homes who were not medical professionals, in the RNs’ work environments^27^. The ability of nurses to perform their duty and roles is highly influenced by their work environment^8^. In other words, an extremely unfavorable work environment due to working long hours, lack of support, being isolated, feeling overloaded, and feeling worthless leads to nurses’ exhaustion, missed care, and poor care quality^18,23,26–28^.

Therefore, our findings suggest that creating a good work environment is critically important to support RNs in maintaining residents’ safety and in providing high quality care in nursing homes. Flynn et al.^25^ claimed that modifiable characteristics of the work environment can be improved through administrative policy strategies. To create a good work environment in nursing homes, Flynn et al.^25^ proposed strategies such as providing managerial training programs to front-line nurse managers and encouraging participation in such programs, increasing RNs’ opportunities to participate in the decision-making process of their institutions, supporting the consistency of patient care assignments, increasing opportunities for continuing education for RNs, and guaranteeing that nursing administrators are accessible, easy to find, and responsive to the concerns of nurses while ensuring the high quality of nursing care. In the case of South Korea, along with these strategies, further efforts are needed, such as providing and disclosing working conditions and wage guidelines in the long-term care service website to induce appropriate levels of remuneration for RNs, and differentiating the scope and roles of RN and CNAs, as well as establishing a work linkage and supervision system between the two roles at institutional and policy levels^27^.

Third, the education levels of the RNs showed a significant association with the incidence rates of pressure ulcers. Few studies appear to have been conducted to examine the association between RN’s education levels and adverse events in nursing homes. Therefore, this study sought to compare its findings with those of studies performed in acute care settings. While a previous study has indicated that higher levels of education among RNs are associated with lower risks of failure to rescue or of mortality^29^, nursing education levels have been found to be inconsistently related to the occurrence of other adverse events in a systematic review^30^. Nevertheless, this study’s findings are consistent with previous research findings concerning other adverse events, where it has been found that hospitals with a higher percentage of RNs with bachelor’s or higher degrees had lower decubitus ulcer rates^31^ and that RNs with a bachelor’s degree were 35% more likely than those with diplomas to have observed an adverse drug reaction^32^.

To promote pressure ulcer prevention, a range of observations and supervision activities, as well as interventions including evaluating risks, assessing skin condition, changing patients’ positions, providing good nutrition and water, and using pressure redistributing devices and skin protection creams are required^33^. The findings of this study indicate that appropriate RN education is crucial to the effectiveness of nurse surveillance^30^. The percentage of RNs with bachelor’s or higher degrees working in nursing homes (42.1%) was found to be less than that of RNs working in acute care settings (73.3%) in a South Korean nationwide health and medical personnel survey^34^. Additionally, RN recruitment strategies in South Korean nursing homes have been largely focused on employing RNs who had taken a long break from the profession^35^. According to the 2018 database by the National Health Insurance Service of Korea^35^, while 67.9% of RNs in acute hospitals were in their 20 s and 30s^34^, 63.3% of the RNs in nursing homes were in their 40 s and 50 s; the RNs belonging to the older age groups inevitably cited reemployment after a long career interruption. In addition, they are the generations who learned in an era where three-year and four-year nursing education coexisted before the higher education law was passed in 2011^22^. Therefore, sufficient professional training should be provided to reequip those RNs who reenter the profession after a significant break and continuing education programs should be strengthened. This study’s finding highlights the necessity for more active recruiting and for retaining more highly educated RNs in nursing homes. Education programs to develop nursing competency for older adults should be robustly implemented so that nursing students can consider long-term care employment or entrepreneurship^35^. Furthermore, it is necessary to improve the work environment to recruit and retain more RNs with bachelor’s degrees in nursing homes.

The education level of RNs was not found to significantly influence other adverse events in this study. In this regard, Audet et al.^30^ explained that few well-validated risk adjustment models account for patients’ baseline risk, with only a limited number of accurate and efficient methods available to measure adverse events. Therefore, reexamining the association between the RNs’ education level and adverse events is recommended, involving appropriate adjustments related to the risks faced by those in resident care, and for the development of a more accurate detection system for adverse events in the future.

This study had some limitations. First, data were collected using questionnaires dependent on nurse self-reports because of the absence of a recording and reporting system concerning adverse events and health outcomes among nursing home residents in South Korea; therefore, reporting bias could not be excluded. To address this limitation in future, it is necessary to develop and manage national electronic recording and reporting systems concerning health outcomes including adverse events for residents in nursing homes in South Korea. Second, residents’ baseline risk factors were not considered, such as physical and cognitive functions, in connection with incidences of adverse events. Finally, as this study involved a cross-sectional design, the longitudinal effects of RN staffing levels, their work environment, and their education levels on residents’ outcomes were not assessed.

## Conclusions

This study showed that RN staffing levels were an important factor affecting the incidences of adverse events in nursing homes. Additionally, the work environment of nurses was found to a key factor, with the likelihood of adverse events being reduced in a good working environment. These findings provide evidence-based support for ensuring appropriate nurse staffing levels and work environments in relation to positive nursing home residents’ health outcomes and accord with the findings of studies performed in other countries. Previous studies in South Korea have not analyzed the effects of education level on adverse events. Nevertheless, education levels affect the quality of care provided by RNs in nursing homes, and this study showed that RNs with higher education levels were associated with lower incidences of pressure ulcers. To improve the health outcomes of residents in nursing homes, considerable attention and efforts are needed to strengthen the workforce of the RNs as well as to improve the nursing work environment at institutional and policy levels.

## Data Availability

The datasets generated during and/or analyzed during the current study are available from the corresponding author on reasonable request.
